# Molecular footprints of the Holocene retreat of dwarf birch in Britain

**DOI:** 10.1111/mec.12768

**Published:** 2014-05-16

**Authors:** Nian Wang, James S Borrell, William J A Bodles, Anasuya Kuttapitiya, Richard A Nichols, Richard J A Buggs

**Affiliations:** * School of Biological and Chemical Sciences, Queen Mary University of LondonLondon, E1 4NS, UK; † Highland BirchwoodsLittleburn, Munlochy, Ross-shire IV8 8NN, UK

**Keywords:** climate change, ecological niche modelling, hybridization, introgression, polyploidy

## Abstract

Past reproductive interactions among incompletely isolated species may leave behind a trail of introgressed alleles, shedding light on historical range movements. *Betula pubescens* is a widespread native tetraploid tree species in Britain, occupying habitats intermediate to those of its native diploid relatives, *B. pendula* and *B. nana*. Genotyping 1134 trees from the three species at 12 microsatellite loci, we found evidence of introgression from both diploid species into *B. pubescens*, despite the ploidy difference. Surprisingly, introgression from *B. nana*, a dwarf species whose present range is highly restricted in northern, high-altitude peat bogs, was greater than introgression from *B. pendula*, which is morphologically similar to *B. pubescens* and has a substantially overlapping range. A cline of introgression from *B. nana* was found extending into *B. pubescens* populations far to the south of the current *B. nana* range. We suggest that this genetic pattern is a footprint of a historical decline and/or northwards shift in the range of *B. nana* populations due to climate warming in the Holocene. This is consistent with pollen records that show a broader, more southerly distribution of *B. nana* in the past. Ecological niche modelling predicts that *B. nana* is adapted to a larger range than it currently occupies, suggesting additional factors such as grazing and hybridization may have exacerbated its decline. We found very little introgression between *B. nana* and *B. pendula*, despite both being diploid, perhaps because their distributions in the past have rarely overlapped. Future conservation of *B. nana* may partly depend on minimization of hybridization with *B. pubescens*, and avoidance of planting *B. pendula* near *B. nana* populations.

## Introduction

Patterns of genetic variation within and among present day species provide evidence about past population dynamics and demographics. However, interpretation of such genetic evidence is difficult, with multiple historical scenarios potentially explaining the same data. A recent example is the observation of Neanderthal-like genetic variants in modern human population of Eurasia. This observation has been variously explained by: a single hybridization event (Green *et al.* 2010), ancient population structure (Durand *et al.* 2011; Eriksson & Manica 2012; Sankararaman *et al.* 2012; Yang *et al.* 2012) or hybridization at a moving front as modern humans invaded Eurasia (Currat & Excoffier 2011). Such ambiguous situations may be to some extent resolved by additional data sources such as other genetic markers, sample areas, taxa or fossils (Wall *et al.* 2013). Multiple data sets from exemplar case studies may aid the interpretation of other systems where only a single set of genetic data is available (Buggs 2007).

One major historical influence on patterns of extant genetic variation is past climate change. Gradients of genetic diversity within species in temperate regions, and correlation of gene phylogenies with geography, can be interpreted as legacies of postglacial recolonization with climate warming (Hewitt 1999; Avise 2000; Petit *et al.* 2003). More detailed evidence about species range shifts in response to climate change may be provided by patterns of genetic exchange between closely related species that meet at hybrid zones (Buggs 2007): specifically, neutral alleles are expected to introgress from a retreating species into an expanding species, leaving behind a molecular footprint of hybrid zone movement (Buggs 2007; Currat *et al.* 2008; Scriber 2011). Whilst this is a potentially sensitive way of tracing past range shifts, genetic patterns alone may not be sufficient to draw firm conclusions, as illustrated by the case of Neandertals and modern humans mentioned above.

Many tree species hybridize extensively with local relatives, making them good study systems for examining patterns of introgression as a consequence of climate change (Petit *et al.* 1997). There is much evidence that tree species have shifted their latitudinal and altitudinal ranges in response to climate change (Davis & Shaw 2001), and this process is ongoing as the climate warms (Chen *et al.* 2011). Evidence for this comes from pollen records (Huntley & Birks 1983), population genetic variability (Petit *et al.* 2003) and phylogenies (Himes *et al.* 2008). In areas bounded by inhospitable habitat, some tree species can only respond to climate change by contracting, rather than shifting their ranges leading to the possibility of local extinction (Zhu *et al.* 2012).

In this study, we set out to test the hypothesis that the decline of a cold-adapted tree species during Holocene climate warming in Britain could be traced in patterns of introgression of its alleles into a closely related tree species that is currently widespread. To aid the interpretation of these introgression patterns, we also analysed patterns of introgression between the widespread species and another close relative with which it is commonly sympatric. We chose a study system with a good fossil record, a well-characterized ecology, and evidence for frequent hybridization. This system is the *Betula* species of Britain. The genus *Betula* (birches) consists of wind-pollinated tree species, which frequently hybridize (Nagamitsu *et al.* 2006; Thórsson *et al*. 2010).

In Britain, there are three native *Betula* tree species: tetraploid *Betula pubescens* and diploids *B. pendula* and *B. nana*. *Betula pubescens* (downy birch) and *B. pendula* (silver birch) are common, widespread and often sympatric or parapatric, with the former adapted to wetter and colder habitats than the latter (Atkinson 1992). *Betula pubescens* is thus more concentrated in northern and western parts of Britain, whereas *B. pendula* is more common in south and east (Gimingham 1984). The two species are hard to distinguish morphologically as there is a continuum of variation between them (Brown & Tuley 1971; Atkinson & Codling 1986). Initially, both were treated as *B. alba* (Linnaeus 1753) and were split later partly due to the difference in ploidy level (Brown & Aldawoody 1979; Gill & Davy 1983; Brown & Williams 1984). Hybrids between the two are thought to occur in many areas in the British Isles, some of which are fully fertile (Stace 2010). Bidirectional gene flow has occurred between *B. pendula* and *B. pubescens*, in Scandinavia and western Russia, but with a bias towards gene flow from *B. pendula* to *B. pubescens* (Palmé *et al*. 2004), perhaps because gene flow is easier from a diploid to a tetraploid than vice versa (Stebbins 1971).

*Betula nana* (dwarf birch) grows up to only one metre in height and is nationally scarce in Britain, mainly restricted to the Scottish Highlands in fragmented populations (Aston 1984). It is widespread in subarctic tundra and subalpine areas of more northerly countries (DeGroot *et al.* 1997). In Scotland*, B. nana* is under active conservation management by organizations such as Trees for Life and Highland Birchwoods. Hybrids between *B. nana* and *B. pubescens* have been recorded in the British Isles (Kenworthy *et al.* 1972; Crawford 2008; Stace 2010). In Iceland, such hybrids have been confirmed using flow cytometry (Anamthawat-Jónsson *et al*. 2010), morphology (Elkington 1968; Thórsson *et al*. 2007), cytogenetics (Anamthawat-Jónsson & Thórsson 2003) and genetic markers (Thórsson *et al*. 2001; Palmé *et al*. 2004). The morphology of ancient pollen of *Betula* species in European pollen cores suggested that hybridization between *B. nana* and *B. pubescens* has taken place throughout the Holocene (Blackburn 1952; Caseldine 2001). Due to the different cold tolerances of the three *Betula* species of Britain, we would expect *B. nana* to be the first colonist of areas coming available after glaciation, followed with climate warming by *B. pubescens* and finally by *B. pendula*.

Therefore, we considered the *Betula* species of Britain to be a good study system to rigorously test the hypothesis that the decline of a species with climate warming could be traced in patterns of introgression of its alleles into a closely related species. We surveyed genetic variation at 12 microsatellite loci in 78 populations of *B. pubescens* and 10 populations of *B. nana* in Britain, hypothesizing that a trail of alleles from *B. nana* would be found in *B. pubescens* populations far south of the current range of *B. nana*. We expected overall rates of introgression to be low (even if rates of hybridization were high) due to the ploidy difference between the two species, which should result in partial reproductive isolation between them. As a point of comparison in interpreting our results, we also genotyped 32 populations of *B. pendula*. Because *B. pendula* is morphologically similar and broadly sympatric with *B. pubescens,* we hypothesized that more gene flow would have occurred between these two species. We also used ecological niche modelling (ENM) and pollen records to infer the current potential distribution of *B. nana* and its past distribution, to provide ecological and historical context for the interpretation of our genetic data.

## Materials and methods

### Sampling and morphological identification

Leaf and twig samples were collected from naturally occurring *Betula* populations across Britain between April 2010 and August 2013. Samples were pressed and dried in a plant press. Species were identified based on leaf morphology according to the standard guide for UK birch identification (Rich & Jermy 1998), including the Atkinson discriminant function to seek to distinguish between *B. pendula* and *B. pubescens* (Atkinson & Codling 1986). In total, 1134 *Betula* samples were collected from 120 populations. Of these, 120 samples were provisionally identified as *B. nana*, 169 as *B. pendula* and 845 as *B. pubescens* (including some of possible hybrid origin). Figure S1 (Supporting information) shows a representative subset of leaves from the three study species. Three known F_1_ hybrid individuals were also examined, two *B. nana* × *B. pubescens* and one *B. nana* × *B. pendula*, which were grown from seed at Queen Mary University of London.

### Microsatellite genotyping

Genomic DNA was isolated from dried cambial tissue or leaves following a modified cetyltrimethylammonium bromide (CTAB) protocol (Wang *et al.* 2013). The isolated DNA was assessed with a Nanovue spectrophotometer (GE Healthcare, UK) and a 1.0% agarose gel. The DNA was diluted to a final concentration of 5–20 ng/μL for subsequent use. A subset of microsatellite loci developed for *B. pendula* (Kulju *et al.* 2004) and *B. pubescens* ssp. *tortuosa* (Truong *et al.* 2005) was used (Table S1, Supporting information). The 5′ terminal of forward primers was labelled with FAM, HEX or TAM. Multiplex PCRs were conducted combining four pairs of microsatellites in each multiplex. In each multiplex reaction, two loci with a significant length difference were labelled using the same dye. The final reaction volume was 7.5 μL, including 3.75 μL QIAGEN Multiplex PCR Master Mix, 0.15 μL of primers (10 μm each in initial volume), 1.55 μL H_2_O and 5–20 ng of DNA dissolved in 1.0 μL TE buffer. Two touchdown PCR programmes (Mellersh & Sampson 1993) were used with differing annealing temperatures according to the primers within each multiplex. For Multiplex 1 and Multiplex 2 (Table S2, Supporting information), an initial denaturation step at 95 °C for 15 min was followed by 28 cycles of denaturation (94 °C for 30 s), annealing (65 °C to 62 °C for 90 s) and extension (72 °C for 60 s) steps, and a final extension step at 60 °C for 30 min. For Multiplex 3 and Multiplex 4 (Table S1, Supporting information), the annealing temperature was from 62 °C to 48 °C, with the remaining steps unchanged. Fragment lengths were determined by capillary gel electrophoresis with capillary sequencer ABI 3730xl (Applied Biosystems). To check the reproducibility of our microsatellite analyses, we selected a subset of 26 individuals, and repeated the microsatellite analyses of these for each individual. The results indicated 100% match in the results, suggesting that our microsatellite analyses are highly reproducible. Alleles were scored using the software genemarker 2.4.0 (Softgenetics) and checked manually.

Three loci with variable flanking regions were genotyped with two sets of primers each to avoid null alleles. One locus, L52, was discarded due to difficulty in reading alleles. Thus, a total of 12 loci were genotyped in our samples. Individuals with more than two missing loci were excluded, resulting in 1134 individuals in the final data set. This data set is available in the Dryad Digital Repository (Wang *et al.* 2014).

### Microsatellite data analysis

Principal coordinates (PCO) analysis of microsatellite data was performed using POLYSAT (Clark & Jasieniuk 2011) implemented in r 2.15.3 (R Core Team 2012), based on pairwise genetic distance calculated by Bruvo's methods (Bruvo *et al.* 2004). POLYSAT is designed to analyse polyploid microsatellite data by assuming that the allele copy number is always ambiguous in any heterozygotes. POLYSAT was also used to transform the multilocus allele phenotype for each individual into binary arrays of the presence or absence of each allele for each individual, and a further PCO analysis was performed using past 1.7.5 (Hammer *et al.* 2001) using pairwise Euclidean distances (Kloda *et al.* 2008).

We also analysed the microsatellite data with a Bayesian clustering approach in structure 2.3.4 (Pritchard *et al.* 2000) to identify the most likely number of genetic clusters (K), to complement the inference of three disjunct clusters from PCO analysis and taxonomic classification. This implements algorithms accounting for genotypic uncertainty arising from copy number variation when the data include polyploid cytotypes. Individuals are assigned to genetic clusters based on multilocus genotypes. Putative hybrids and admixed individuals could be identified as they have fractions of genomes from different genetic clusters. We performed ten replicates (1 000 000 generations and a burn-in of 100 000 for each run) at each value of K from one to five under the admixture model with the assumption of correlated allele frequencies among populations. Individuals were assigned to clusters based on the highest membership coefficient averaged over the 10 independent runs. The *ΔK* was calculated based on the rate of change in the log probability of the data between successive K values (Evanno *et al.* 2005). Replicate runs were grouped based on a symmetric similarity coefficient of >0.9 using the Greedy algorithm in clumpp (Jakobsson & Rosenberg 2007) and visualized in distruct 1.1 (Rosenberg 2004). We chose the optimal value of K based on the PCO analysis and the *ΔK* analysis of the structure outputs.

The slopes of the latitudinal clines in the admixture proportions (the structure values, logit-transformed) were estimated using a mixed effects model, with slope as a fixed effect and population modelled as a random effect, to allow for genetic drift of each population away from the trend. This analysis was implemented using the lme function in r 2.15.3 (Pinheiro & Bates 2000). Despite logit transformation of the proportions, the residuals were slightly asymmetrical so, as an additional test, the null distribution of slopes was estimated by permuting the latitudes among populations and repeating the analysis, using a custom script in r 2.15.3. Our r scripts are available in the Dryad Digital Repository (Wang *et al.* 2014).

Population genetic parameters were calculated for the selected 55 populations with at least eight individuals from each population. These include six *B. nana* populations, 39 *B. pubescens* populations and 10 *B. pendula* populations (Table S3, Supporting information). Pairwise F_ST_ tests based on allele frequency were conducted for these populations in POLYSAT. A matrix of geographical distance was generated based on latitude and longitude in r package ‘fields’ (Furrer *et al.* 2011). A Mantel test with 9999 permutations was conducted in r package ‘ade4’ to test for a significant signal of isolation by distance (Dray & Dufour 2007).

### Distribution range modelling

To model the potential distribution range of the three *Betula* species in Britain, all available occurrence records for the three species were organized into a single database from a number of sources (Botanical Society of the British Isles, National Biodiversity Network, Highland Birchwoods and Scottish Natural Heritage), resulting in 48 164 records. The data were filtered to include only complete records with a spatial resolution <1 km and dated post-1950 to remain consistent with available environmental data; this resulted in 11 879 records. Twenty-two bioclimatic variables were considered as possible predictors for *Betula* species distribution. These included 19 bioclimatic variable layers obtained from WorldClim (http://www.worldclim.org) (Hijmans *et al.* 2005); elevation data, also obtained from WorldClim; and soil type and peat depth (where >2 m) variables (categorical) obtained from the european soil database version 2 (http://eusoils.jrc.ec.europa.eu). All layers were resampled to 1 km resolution and clipped to include only the British Isles using Environmental Systems Research Institute's arcgis version 10. Modelling was conducted in maxent version 3.3 (Phillips *et al.* 2004, 2006), a maximum entropy-based machine-learning programme that estimates the probability distribution for species occurrence, based on environmental predictors and presence-only data. We ran maxent under default settings, with 10 subsampled replicated runs, a limit of 5000 iterations and 25% of the data partitioned for testing of the model. maxent was used to calculate the area under the curve (AUC) averaged over the replicate runs, to allow comparison of model performance between the study species. Resulting values range from 0.5 (random) to 1.0 (exact match). The resulting potential species distribution map was then opened and manipulated in arcgis. Thresholds probabilities for species presence are unknown, thus the resulting values ranging from 0 to 0.88 and were arbitrarily regrouped into six classes: 0–0.15, 0.16–0.30, 0.31–0.45, 0.46–0.60, 0.61–0.75 and 0.76–0.90.

Niche overlap between species was measured using Schoener's D (Schoener 1968), and the I statistic (Warren *et al.* 2008), calculated in enmtools version 1.4.3 (Warren *et al.* 2010). Similarly, species range overlap was also tested in enmtools version 1.4.3, over a range of manually defined presence probability thresholds to explore the characteristics of the data. We chose a conservative value of 0.45, although we note that the comparative relationships between the three species remain consistent over a broad range.

### Pollen record gathering

To build a picture of the past distribution of these species in the UK, we examined pollen records of *Betula* species in the European Pollen Database (EPD, http://www.europeanpollendatabase.net/data/). For some pollen cores, palaeobotanists have identified pollen type to the species level, whereas, others are identified at the genus level only. We mapped these pollen sites using coordinates given in the EPD. For eight pollen sites, coordinates are not given in the EPD, so we mapped the sites according to the geographical descriptions given in the original literature. The detailed pollen records are listed in Table S2 (Supporting information).

## Results

### Microsatellite analysis

**Figure 1:**
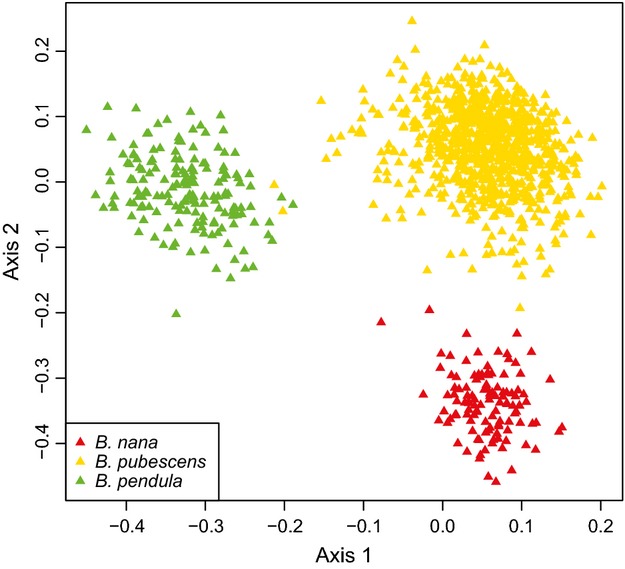
Principal coordinates (PCO) analysis of microsatellite genotypes in *B. nana*,*B. pubescens* and *B. pendula* populations sampled, based on Bruvo's genetic distance.

Broad characterization of genetic diversity among the three *Betula* species was conducted with principal coordinates (PCO) analysis. The Bruvo's genetic distances of all 1134 individuals were calculated and scaled. The first axis separated *B. pendula* from a cluster of *B. pubescens* and *B. nana*, and the second axis separated *B. nana* from *B. pubescens* and *B. pendula*. Thus, three distinct clusters corresponded to *B. nana*,*B. pubescens* and *B. pendula* (Fig.1). The PCO analysis of these individuals based on pairwise Euclidean distances showed a similar pattern (Fig. S2, Supporting information).

**Figure 2:**
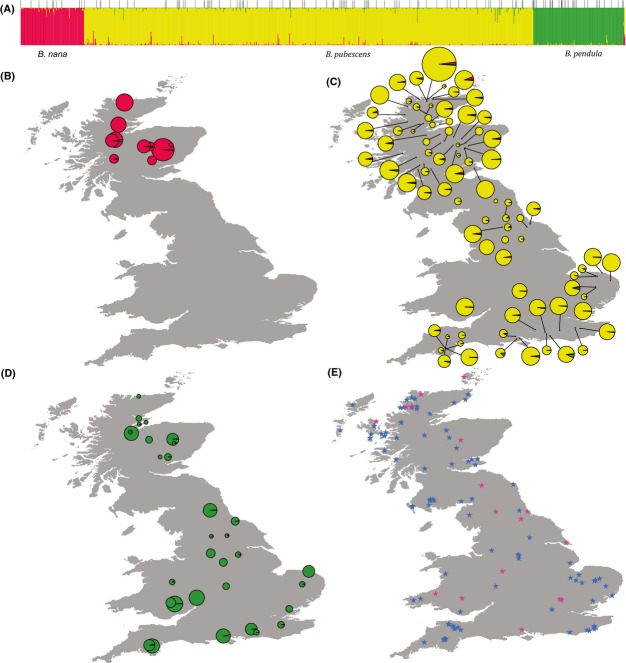
Genetic admixture among the three native *Betula* species in Britain, with locations of populations tested, and pollen fossil sites. (A) Sharing of microsatellite alleles among the three species *B. nana*,*B. pubescens* and *B. pendula* shown as a structure plot with K = 3 corresponding with the three species. Within each species grouping, populations are ordered by latitude, with more northerly populations to the left-hand side. Thin vertical lines above the structure plot indicate population divisions. Three known F_1_ hybrid seedlings are shown on the far right: *B. nana* × *B. pendula*,*B. nana* × *B. pubescens* and *B. nana* × *B. pubescens*, respectively. (B–D) The locations of the sampled populations of *B. nana*,*B. pubescens* and *B. pendula*, respectively: pie charts show the mean proportion of individual genotypes in each population assigned to a particular lineage by structure, and pie chart size is proportional to the sample size for each population. The centre of pie charts represents approximately its sampling locality unless the pie chart is connected to its sampling locality by a straight line. (E) Pollen sites of *Betula* species across Britain. Red stars represent the pollen sites of *B. nana* and *B*. *nana,* and blue stars represent the pollen sites of *Betula* likely to be *B. pubescens* and *B. pendula*.

**Figure 3:**
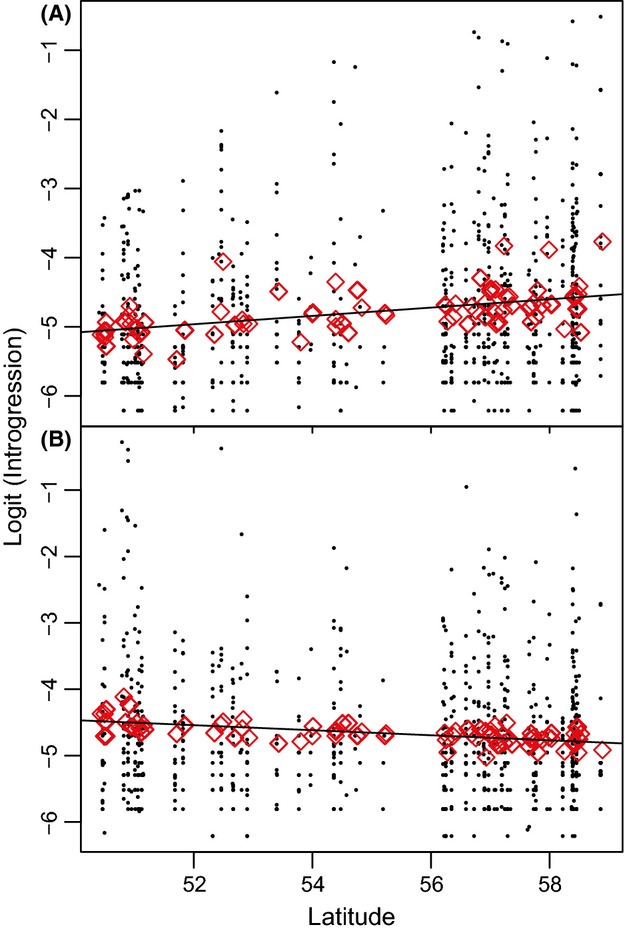
Clines of *B. nana* and *B. pendula* admixture in *B. pubescens* populations. The latitude of each sample populations is shown on the horizontal axis, and logit-transformed structure admixture proportions for each *B. pubescens* individual are shown as circles. Red diamonds represent the value for each *B. pubescens* population fitted by the mixed effects model. (A) The cline of *B. nana* admixture into *B. pubescens* populations, which showed a significant positive correlation with latitude (*P *=* *0.0045). (B) The cline of *B. pendula* admixture into *B. pubescens* populations, which showed a significant negative correlation with latitude (*P *=* *0.0166).

Genetic admixture among species within individuals was examined with Bayesian analysis using structure under the admixture model. Analysis was conducted assuming three populations (K = 3) based on clear clustering in the PCO distribution, corroborated by the *ΔK* criterion (Fig. S5A, Supporting information). The estimated admixture between *B. pendula* and *B. nana* was negligible (Fig.2), but admixture was inferred between *B. pubescens* and *B. nana* and also between *B. pubescens* and *B. pendula* (Fig.2). Higher levels of admixture from *B. nana* to *B. pubescens* were found in the north than in the south of Britain. The cline of *B. nana* admixture in *B. pubescens* populations was positively correlated with latitude (Fig.3A, *P* = 0.0045). Conversely, the cline of *B. pendula* admixture in *B. pubescens* was negatively correlated with latitude (Fig.3B, *P* = 0.0166).

Significant isolation-with-distance was detected among *B. nana* populations (Fig. S3, Supporting information, Mantel test, *r* = 0.7035, *P *=* *0.0086) and among *B. pubescens* populations (Fig. S3, Supporting information, Mantel test, *r* = 0.1384, *P *=* *0.0093), but not among *B. pendula* populations (Fig. S3, Supporting information, Mantel test, *r* = −0.0418, *P *=* *0.5709). Genetic differentiation between *B. nana* and *B. pendula* was higher than between *B. nana* and *B. pubescens*, and between *B. pubescens* and *B. pendula* (Fig. S4, Supporting information). Genetic structure was detected among *B. nana* populations, but not among either *B. pubescens* or *B. pendula* populations when the three species were analysed independently with the admixture model (Fig. S6, Supporting information).

### Model-based prediction of past distribution ranges

**Figure 4:**
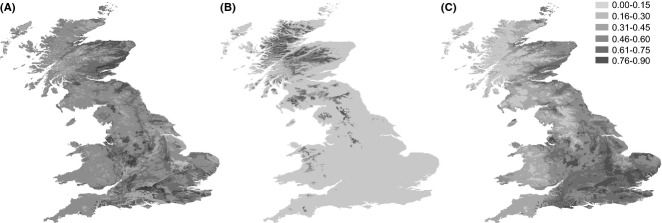
Ecological niche model predicted distribution British ranges for (A) *B. pubescens* (B) *B. nana* and (C) *B. pendula*. At an occurrence probability threshold of 0.45, range overlap is as follows: *B. pubescens* and *B. pendula* (73%); *B. pubescens* and *B. nana* (5%); *B. nana* and *B. pendula* (0%).

Ecological niche models constructed with maxent from species occurrence records, performed well for *B. nana* (AUC = 0.959, SD = 0.018) and were satisfactory for *B. pendula* (0.723 ± 0.009) and *B. pubescens* (0.645 ± 0.008). The most important environmental predictors were soil type and annual mean temperature, with the exception of *B. nana* for which altitude was of primary importance. The results suggest that suitable habitats for *B. nana* may currently exist in large areas in the Scottish Highlands, SW England, Wales, middle and North England (Fig.4): an area larger than the area currently occupied by *B. nana*. Suitable habitat for *B. pubescens* and *B. pendula* appears widespread in Britain, the most suitable habitat for *B. pendula* being towards the south and east, and suitable habitat for *B. pubescens* being widespread. Analysis of pairwise niche overlap revealed considerable similarity between *B. pubescens* and *B. pendula* niches (Schoener's D = 0.82, I = 0.97). There was substantially less overlap when comparing *B. nana* with *B. pubescens* (D = 0.25, I = 0.58) and *B. pendula* (D = 0.18, I = 0.48). Range overlap analysis at a conservative occurrence probability threshold (0.45) identified extensive overlap between *B. pubescens* and *B. pendula* (73%) and small overlap between *B. pubescens* and *B. nana* (5%), but no range overlap between *B. nana* and *B. pendula*. Suitable habitats for *B. nana* either overlap with or are surrounded by suitable habitats for *B. pubescens* (Fig.4).

### Pollen records

*Betula* ssp. pollen was found recorded for 112 British sites of preserved pollen in the European Pollen Database. The majority most likely represent *B. pubescens* and *B. pendula*, which produce abundant pollen, but 13 sites contained pollen identified as *B. nana* pollen and four contained pollen identified as ‘*B*. *nana*’ (Fig.2E; Table S2, Supporting information). These included sites in the south that are outside the current range of *B. nana* suggesting a much more southerly distribution of *B. nana* in the past. These pollen records provide us with a longer-term view of the past distribution ranges of *B. nana* than herbarium collections.

## Discussion

Allele sharing among closely related species may occur for a variety of reasons: (A) shared alleles may have been inherited from a polymorphic common ancestor due to incomplete lineage sorting, (B) convergent mutations may have caused the same alleles to have arisen independently in different species, (C) alleles may have moved from one species to another via introgressive hybridization within a framework of stable species' ranges, perhaps assisted by selection, (D) alleles may have moved via introgressive hybridization where neutral gene flow has been increased by the retreat of the range of one species and the concomitant expansion of the range of the species with which it can hybridize. We consider that in the present study, the balance of evidence points towards (D).

Although incomplete lineage sorting (A) may be frequent among tree species due to their large effective population sizes and long generation times (Bouillé & Bousquet 2005; Chen *et al.* 2010), this seems an unlikely explanation for the patterns of allele sharing observed between *B. nana* and *B. pubescens*, because we find a gradient of *B. nana* alleles that increases closer to the current location of *B. nana* populations in the north. If the loci in the study were neutral with respect to selection, which is expected of microsatellite alleles, then incomplete lineage sorting would not be expected to give a geographic signal (Barton 2001).

Convergent mutations (B) also seem an improbable explanation. Convergence is intrinsically unlikely at neutral loci, and if it did occur, it would be expected to yield symmetric allele sharing between *B. pubescens* and *B. nana*. However, we observe a pattern of asymmetric allele sharing between *B. pubescens* and *B. nana* (Fig.2).

The pattern we observe is therefore likely to be caused by hybridization. *Betula nana* and *B. pubescens* currently have parapatric distributions and often occur close together in natural environments. Several putative hybrids have been noted by taxonomists in Scotland (Kenworthy *et al.* 1972), and extensive hybridization and gene flow have been shown to occur between the two species in Iceland (Anamthawat-Jónsson & Thórsson 2003; Maliouchenko *et al.* 2007), Scandinavia and Russia (Maliouchenko *et al.* 2007). However, the pattern of introgression that we observe is unlikely to have been caused (C) simply by spread of alleles from the current distribution range of *B. nana*. High genetic differentiation and significant isolation-with-distance (Fig. S3, Supporting information) among *B. nana* populations suggest that *B. nana* has a low capacity for gene flow, as is to be expected for a dwarf tree producing small amounts of pollen and seed compared to its larger tree relatives (Bradshaw 1981). Also, because microsatellites markers are expected to be neutral to selection, the presence of *B. nana* alleles in occasional *B. pubescens* populations far from the present range of *B. nana* in the middle of Britain is unlikely to have been caused by natural selection.

The observed level of introgression from *B. nana* to *B. pubescens* is not less than the level of introgression we observe from *B. pendula* to *B. pubescens* (Student's *t*-test, *t* = 0.082, *P *=* *0.934). This is surprising given that *B. pendula* is a tree that disperses more pollen than *B. nana* and frequently occurs in sympatry with *B. pubescens* in much of its British range (Atkinson 1992). Given that *B. nana* and *B. pendula* are diploid with the same chromosome number, they are unlikely to differ in chromosomal postzygotic reproductive isolation with tetraploid *B. pubescens*. Hybrids between *B. pendula* and *B. pubescens* have been recorded in the UK (Brown *et al.* 1982), and a study of chloroplast introgression in Scandinavia and western Russia found higher rates of introgression between *B. pendula* and *B. pubescens* than between *B. nana* and *B. pubescens* (Palmé *et al*. 2004). The fact, therefore, that we find similar introgression from *B. nana* to *B. pubescens* and from *B. pendula* to *B. pubescens* requires an explanation.

The most likely explanation of the pattern observed in this study is (D) that we are seeing a trail of introgression resulting from past retreat of the range of *B. nana* accompanied by the northwards expansion of the range of *B. pubescens*. This could explain the high level of introgression found relative to *B. pendula*–*B. pubescens* introgression and the geographic pattern of introgression observed. This hypothesis fits with the fact that fossils of *B. nana* and *B*. *nana* pollen are distributed across Britain (Fig.2E) showing a larger and more southerly range in the past. Both genetic and fossil evidence therefore point to the northwards movement of the range of *B. pubescens* in the UK, at the expense of *B. nana*, with some hybridization occurring between them during this expansion/retreat, leaving a molecular footprint.

What caused this expansion of *B. pubescens* at the expense of *B. nana*? The fact that *B. nana* pollen is found outside the current environmental niche range of *B. nana* suggests that past climate change has played a major role in the species' decline, specifically climate warming in the Holocene after the last glacial maximum. But the fact that *B. nana* is currently more restricted in its range than the area that it is adapted to according to the ENM suggests that other factors may also have contributed to the decline of *B. nana*, such as overgrazing by sheep and deer (Tanentzap *et al.* 2013), and burning of moorland for grouse shooting (DeGroot *et al.* 1997). Our study suggests a further contributing factor may be pollen swamping of *B. nana* by *B. pubescens*, reducing the production of fertile *B. nana* offspring in *B. nana* populations. The low levels of introgression found in this study support the pollen-swamping hypothesis: due to the ploidy difference between *B. nana* and *B. pubescens*, we expect most hybrids to be sterile, so only a minority of hybrids formed will be capable of contributing to introgression between the two species. Therefore, the small amount of introgression we observe between *B. nana* and *B. pubescens* suggests that large numbers of hybrids have been formed, as has been found in Icelandic populations of *B. nana* and *B. pubscens* where up to 10% of trees may be hybrids (Anamthawat-Jónsson & Tómasson 1999; Anamthawat-Jónsson & Thórsson 2003). Furthermore, the asymmetric pattern of gene flow that we observe suggests that on the rare occasions when hybrids are capable of backcrossing, they do so mainly with *B. pubescens*, rather than *B. nana*. This, and the fact that *B. pubescens* is a tree with far greater pollen dispersal ability than *B. nana*, suggests that *B. nana* ovules may be frequently fertilized by *B. pubescens* pollen. Thus, reproduction of *B. nana* may be reduced by the production of (mainly sterile and nonbackcrossing) hybrids with *B. pubescens*. Such a dynamic has been shown to occur in a hybrid zone between diploid and hexaploid *Mercurialis annua*, where the hexaploid form is apparently being eliminated by the diploid form due to pollen swamping and the production of sterile hybrids (Buggs & Pannell 2006). Even when hybrids are not mainly sterile, pollen swamping can still contribute to the advance of one species' range at the expense of another, for example, pollen swamping of *Quercus robur* by *Q. petraea* seems to assist the latter in invading the range of the former (Petit *et al.* 2004).

We find very little introgression between *B. nana* and *B. pendula*, despite the fact that a reproductive barrier due to ploidy does not separate them. While we do not know whether other reproductive barriers separate them, we have found diploid hybrids when growing up seeds collected from *B. nana* populations in Scotland, in an area recently planted with *B. pendula* in afforestation, suggesting that *B. nana*—*B. pendula* hybrids do form in Scotland. The most probably explanation for the lack of introgression between the two species in our study is the disjunct nature of their natural distributions: the environmental niches of the two rarely overlap (Fig.4). *Betula nana* is adapted to cold and wet habitats (DeGroot *et al.* 1997), whereas *B. pendula* prefers warm and dry habitats (Gimingham 1984). *Betula nana* commonly grows above the treeline, whereas *B. pendula* grows in regions with low altitude usually below a few hundred metres (Gimingham 1984). A 6-year study in Sweden showed the germination rates of *B. pendula* seeds to decrease strongly with altitude (Holm 1994). Maintenance of the geographical separation between *B. nana* and *B. pendula* may be a key to preventing future hybridization between them.

We conclude that a balance of evidence from both genetic data and fossils suggests that a zone of hybridization between *B. nana* and *B. pubescens* moved northwards through the UK since the last glacial maximum, leaving behind a footprint of introgressed genes in the genome of *B. pubescens*. Though likely to have been mainly driven by climate change, the decline of *B. nana* may have been exacerbated by hybridization with *B. pubescens*. Today, *B. nana* is nationally scarce in Britain and under active conservation management. Successful conservation of *B. nana* may partly depend on minimization of future gene flow from *B. pubescens*. However, a bigger threat may be hybridization with *B. pendula*; although there appears to have been little hybridization between *B. nana* and *B. pendula* in the past, this may be due to ecological separation rather than reproductive incompatibility (Wilsey *et al.* 1998), and planting of *B. pendula* saplings in areas where *B. pendula* could not establish from seeds could cause a new anthropogenic threat to the reproduction of *B. nana*.

## Notes

N.W. and R.J.A.B. designed research; N.W., J.S.B., A.K., W.J.A.B. and R.J.A.B. performed research; N.W., J.S.B. and R.A.N. analysed data; and N.W., J.S.B and R.J.A.B. wrote the paper.

## Data accessibility

Microsatellite data and R scripts used in data analyses are available in the Dryad Digital Repository, doi:10.5061/dryad.mt5sj. Herbarium sheets of specimens will be deposited in the Natural History Museum Herbarium, London. The species occurrence records data used in the distribution range modelling were from: Botanical Society of the British Isles (http://www.bsbi.org.uk), National Biodiversity Network (https://data.nbn.org.uk/), Highland Birchwoods (http://www.highlandbirchwoods.co.uk) and Scottish Natural Heritage (http://www.snh.gov.uk). The bioclimatic and elevation data used in the distribution range modelling are available from WorldClim (http://www.worldclim.org) and the soil data from the European Soil Database version 2 (http://eusoils.jrc.ec.europa.eu). The pollen records used from the European Pollen Database (http://www.europeanpollendatabase.net) and published studies are shown in Table S2 (Supporting information). The population sample site locations from this study are listed in Table S3 (Supporting information).

## Supporting Information

Table S1 Summary of the 16 polymorphic microsatellite loci multiplexed for all samples. **Table S2** Summary of published pollen records of *Betula* species in the UK. **Table S3** Genetic diversity of 55 populations with over seven samples of the three *Betula* species, based on microsatellites. **Fig. S1** Pairs of leaves from a representative sample of trees used in the present study, showing upper and lower sides. **Fig. S2**PCO analysis based on Euclidean distance. **Fig. S3** Pairwise genetic differentiation (F_ST_) within species. **Fig. S4** Pairwise genetic differentiation (F_ST_) between *B. nana*,*B. pubescens* and *B. pendula*. **Fig. S5** (A) The log-likelihood values of each K and *ΔK* values. (B) The structure output at K = 2, 3 and 4. **Fig. S6** The structure output of *B. nana*,*B. pubescens* and *B. pendula* separately, at K = 2, 3 and 4, based on the admixture model.
