# Supplementary material for: Molecular footprints of the Holocene retreat of dwarf birch in Britain
Source: Mol Ecol. 2014 May 16;23(11):2771–82. doi: 10.1111/mec.12768 (PMC4237149; doi:10.1111/mec.12768)

**Online Supplementary Information**

**Molecular footprints of the Holocene retreat of dwarf birch in Britain**

Nian Wang, James S. Borrell, William J. A. Bodles, Ana Kuttapitiya,

Richard A. Nichols, Richard J. A. Buggs

**Table S1.** Summary of the 16 polymorphic microsatellite loci multiplexed for all samples.

**Table S2.** Summary of published pollen records of *Betula* species in UK. Three records (the Upper Teesdale, the Lower Teesdale and Southern England in Table S3) were retrieved from the literature and approximated according to the description and the remaining 109 were obtained from European Pollen Database (EPD).

**Table S3.** Genetic diversity of 55 populations of the three *Betula* species based on microsatellites. Populations with no less than eight samples were chosen.

**Figure S1.** Pairs of leaves from a representative sample of trees used in the present study, showing upper and lower sides. Samples were assigned to the following categories based on morphology and sampling locality of each sample was included in brackets. *Betula nana*: 407 (Desside),410 (Desside) and 398 (Desside); *B. pubescens*: 278_011 (Loch Eriboll (E)), 938 (Ben Loyal)465_002 (Lynemore), 579 (Lairg), 508_014 (Rannoch Moor), 1143 (Newhall), 1010 (Ben Loyal), 1579 (Ben Loyal), 1573 (Ben Loyal), 1008 (Ben Loyal), 13_020 (Beaulieu, New Forest), 2364g (DartmoorHotel), 1009 (Ben Loyal); *B. pendula*: 553 (Birnam), 2363c (DartmoorHotel), 3_008 (Danbury Wood).

**Figure S2.** PCO analysis based on Euclidean distance. Red symbols represent *B. nana*; yellow symbols represent *B. pubescens* and green symbols represent *B. pendula*.

**Figure S3.** Pair-wise genetic differentiation (F_ST_) within species. Red triangle, gold triangle and green triangle represent *B. nana*, *B. pubescens* and *B. pendula* respectively.

**Figure S4.** Pair-wise genetic differentiation (F_ST_) between *B. nana*, *B. pubescens* and *B. pendula*.

**Figure S5.** (A) The log-likelihood values of each K and*ΔK* values. (B) The STRUCTURE output at K = 2, 3 and 4.

**Figure S6.** The STRUCTURE output of *B. nana*, *B. pubescens* and *B. pendula* separately under the model of admixture, at K = 2, 3 and 4.

**Table S1. Details of microsatellite primers used in the present study**

| Locus | Dye | Sequences | Allele Size | Repeat | Multiplex |
| --- | --- | --- | --- | --- | --- |
| L1.1* | HEX | ACGCTTTCTTGATGTCAGCC  TCACCAAGTTCCTGGTGGAT | 168–209 | (GA)_4_AA(GA)_10_ | Multiplex 1 |
| L3.1* | FAM | CTCCTTAGCTGGCACGGAC  CCCTTCTTCATAAAACCCTCAA | 219–241 | (CT)_3_CC(CT)_2_CC(CT)_13_AT(CT)_5_ |  |
| L13.1 | FAM | CACCACCACAACCACCATTA  AACACCCTTTGCAACAATGA | 93–108 | (CA)_3_(GA)_14_ |  |
| L012 | TAM | TGGTTGACGTGACGTTGATT  GGCCCATAGGGAAGATAAGC | 210–222 | (GA)_6_TA(GA)_6_ |  |
| L52* | FAM | AGCTACCCCTGGTCCACTTT  CCGCCTTGGATTTCACTAAA | 250–272 | (CT)_12_ | Multiplex 2 |
| L5.4* | HEX | AAGGGCACCTGCAGATTAGA  AAAATTGCAACAAAACGTGC | 230–262 | (TC)_26_ |  |
| L7.1a | HEX | GTTTTGGGTTTCCACTTCCA  ACTGGTAATACCTTTACCAAGCC | 146–152 | (CT)_12_CCTT(CT)_4_ |  |
| L7.3 | TAM | GGGGATCCAGTAAGCGGTAT  CACACGAGAGATAGAGTAACGGAA | 178–226 | (GT)_18_(GA)_14_ |  |
| L1.1 | FAM | TTTCCAACGCTTTCTTGATG  TGGATAAGGAAGGGCATGTC | 152–206 | (AG)_4_AA | Multiplex 3 |
| L2.3 | HEX | CGGGAAGATATGCAGTGTTT  TTGGCGGGTGAAGTAGAC | 208–252 | (AG)_16_ |  |
| L3.1 | HEX | CACACTGCTGCCTGA  TCATAAAACCCTCAAAGAAT | 134–166 | (CT)_13_A(TC)_6_ |  |
| L021 | TAM | TCTACGCTGTGACCAGTC  AGAATCCTAGCCTTTTCAAT | 168–236 | (CT)_14_ |  |
| L2.5 | FAM | CTATATTGGCTCCAAGCAC  ACACCCACACTGACAGATAA | 94–128 | (CT)_9_ | Multiplex 4 |
| Bo.F330 | FAM | TGGCAGCACGAAAGT  TGGGAATGAGAGAACAAG | 172–210 | (TC)_14_ |  |
| Bo.F394 | HEX | AATGCAGCATCTCTTACC  CACGCAATAATATGGAAA | 128–194 | (TC)_13_ |  |
| L5.4 | TAM | GAAAGCATGAGACCCGTCTT  AACCTAAACAGCCTGCCAAA | 134–188 | (TC)_26_ |  |

*: Discarded due to redundancy or difficulty in allele assigning.

**Table S2. A list of published pollen records across Britain**

| Locality | Latitude | Longitude | Description |
| --- | --- | --- | --- |
| Parsons Park, Hawks and Dozmary Pool, SW England | 50.53333 | -4.6 | *Betula* |
| Broad Down | 50.6125 | -3.962 | *Betula* |
| Winneys Down | 50.62278 | -3.94278 | *Betula* |
| Cut Hill | 50.6275 | -3.98194 | *Betula* |
| Hangingstone Hill | 50.65472 | -3.95694 | *Betula* |
| Stonetor Brook | 50.65556 | -3.91 | *Betula* |
| Caburn | 50.85722 | 0.050556 | *Betula* |
| Pannel Bridge | 50.90583 | 0.675278 | *Betula* |
| Brede Bridge | 50.92861 | 0.599722 | *Betula* |
| Middle North Coombe | 50.93389 | -3.43333 | *Betula* |
| Southern England | 50.93829 | -1.47897 | *B. nana* |
| Little Cheyne Court Walland marsh | 50.96278 | 0.83 | *Betula* |
| Lobbs Bog | 50.97056 | -3.62361 | *Betula* |
| Windmill Rough | 50.97472 | -3.63306 | *Betula* |
| Hares Down | 50.97806 | -3.64389 | *Betula* |
| Exebridge | 51.01722 | -3.51722 | *Betula* |
| Hope farm Walland marsh | 51.01778 | 0.835556 | *Betula* |
| The Dowels Walland marsh | 51.04361 | 0.828056 | *Betula* |
| Horsemarsh Sewer | 51.05194 | 0.826667 | *Betula* |
| Gourte Mires | 51.05444 | -3.67833 | *Betula* |
| Long Breach | 51.06583 | -3.6875 | *Betula* |
| North Twitchen Springs | 51.11722 | -3.82611 | *Betula* |
| Comerslade | 51.12028 | -3.80472 | *Betula* |
| Llanilid | 51.51667 | -3.45 | *Betula* |
| Sladeoak Lane | 51.58528 | -0.51833 | *Betula* |
| Marks Tey | 51.86667 | 0.783333 | *Betula* |
| Esgryn Bottom | 51.87611 | -4.94194 | *Betula* |
| Fishersgreen | 51.9 | -0.217 | *B. nana* |
| Hitchin | 51.917 | -0.267 | *B. nana* |
| Gors Fawr Bog | 51.93167 | -4.71833 | *Betula* |
| traeth mawr, brecon beacons, south Wales | 51.9485 | -3.392 | *B. nana* |
| Shottisham | 52.03333 | 1.4 | *Betula* |
| Mid Wales | 52.09028 | -4.23889 | *B. nana* |
| Willingham Mere | 52.33333 | -2.31667 | *Betula* |
| Hoxne | 52.33556 | 1.2 | *Betula* |
| Redmere | 52.43972 | 0.438056 | *Betula* |
| Hockham Mere | 52.5 | 0.833333 | *Betula* |
| Welney Washes | 52.51667 | 0.25 | *Betula* |
| The Mere Stow Bedon | 52.52944 | 0.873889 | *Betula* |
| Saham Mere | 52.57056 | 1.004444 | *Betula* |
| Sea Mere | 52.57056 | 1.004444 | *Betula* |
| Hobbs Lot March | 52.60167 | 0.071389 | *Betula* |
| Wiggenhall St. Germans | 52.69278 | 0.339444 | *Betula* |
| Llyn Gwernan | 52.72556 | -3.92139 | *Betula* |
| King's Pool | 52.808 | -2.108 | *B. nana* |
| Lade Bank | 53.0725 | 0.057778 | *Betula* |
| Hipper Sick | 53.21667 | -1.58333 | *Betula* |
| Salter Sitch | 53.30167 | -1.56861 | *Betula* |
| Ringinglow Bog | 53.34472 | -1.60139 | *Betula* |
| Chat Moss | 53.467 | -2.45 | *Betula* |
| The Bog | 53.733 | -0.067 | *B.* cf. *nana* |
| Tadcaster | 53.88333 | -1.23333 | *Betula* |
| the lower Teesdale | 54.4847 | -1.5031 | *B. nana* |
| LOONS | 54.60722 | -3.15806 | *Betula* |
| The Upper Teesdale | 54.7163 | -2.312 | *B. nana* |
| Thorpe Bulmer | 54.717 | -1.3 | *B. nana* |
| Houghall Wood | 54.75 | -1.56667 | *Betula* |
| Little Lochans | 54.86667 | -4.98333 | *Betula* |
| Culhorn Mains | 54.89167 | -4.98333 | *Betula* |
| Moss of Cree | 54.92333 | -4.45 | *Betula* |
| Roberthill Farm | 55.05 | -3.41667 | *Betula* |
| Clatteringshaws Loch | 55.06667 | -4.28333 | *Betula* |
| Nick of Curleywee | 55.06778 | -4.4275 | *Betula* |
| Round Loch of Glenhead | 55.08472 | -4.41889 | *Betula* |
| Loch Dungeon | 55.11667 | -4.31667 | *Betula* |
| Cooran Lane | 55.11667 | -4.4 | *Betula* |
| Bigholm Burn | 55.12028 | -3.0725 | *Betula* |
| Beanrig Moss | 55.555 | -2.766 | *B. nana* |
| Bradford Kaims | 55.58889 | -1.76111 | *Betula* |
| Loch Lomond Ross Dubh | 56.08639 | -4.58333 | *Betula* |
| Ross Dubh | 56.08639 | -4.58333 | *Betula* |
| Lundin Tower | 56.215 | -2.96583 | *Betula* |
| Abernethy Forest | 56.23333 | -3.71667 | *Betula* |
| Black Loch | 56.32083 | -3.19472 | *Betula* |
| Creich Castle | 56.38333 | -3.08333 | *Betula* |
| Gallanech Beg | 56.38444 | -5.50139 | *Betula* |
| Pickletillem | 56.4 | -2.9 | *Betula* |
| Le Loch Sunart | 56.66667 | -5.86667 | *Betula* |
| Straloch | 56.75 | -3.56667 | *Betula* |
| Morrone Birkwoods | 57 | -3.433 | *B.* cf. *nana* |
| Loch Cleat | 57.06667 | -6.33333 | *Betula* |
| Loch Einich | 57.08333 | -3.8 | *Betula* |
| Loch Maree | 57.08333 | -5.48333 | *Betula* |
| Loch Meodal | 57.13333 | -5.86667 | *Betula* |
| Loch Tarff | 57.15417 | -4.59583 | *Betula* |
| Lochan coir a' Ghobhainn | 57.18333 | -6.3 | *Betula* |
| Loch Cill Chriosd | 57.2 | -5.96667 | *Betula* |
| Loch Ashik | 57.25 | -5.83333 | *Betula* |
| Allt na Feithe Sheilich | 57.31667 | -3.9 | *Betula* |
| Teanga | 57.31917 | -7.28472 | *Betula* |
| Loch Fada | 57.45 | -6.2 | *Betula* |
| Loch Clair | 57.55889 | -5.34361 | *Betula* |
| Loch a'Chroisg | 57.56833 | -5.32778 | *Betula* |
| Loch Mealt | 57.6 | -6.133 | *B.* cf. *nana* |
| Coire Bog | 57.85 | -4.41667 | *Betula* |
| Loch Craggie | 58.003 | -4.84333 | *Betula* |
| Badentarbat | 58.03583 | -5.36611 | *Betula* |
| Loch Borralan | 58.0441 | -4.99917 | *Betula* |
| Druim Bad a'Ghaill | 58.0528 | -5.26778 | *Betula* |
| Loch Sionascaig | 58.061 | -5.175 | *B. nana* |
| Strath Oykell | 58.0739 | -4.83361 | *Betula* |
| Cam Loch | 58.083 | -5 | *B. nana* |
| Little Loch Roag | 58.13333 | -6.88333 | *Betula* |
| Wag | 58.2 | -3.65 | *Betula* |
| Whittington Graeme | 58.2 | -3.65 | *Betula* |
| Duartbeg | 58.29611 | -5.16306 | *Betula* |
| Ellanmore | 58.3 | -3.28333 | *Betula* |
| Lochan an Smuraich | 58.321 | -4.894 | *Betula* |
| Loch Laxford | 58.36667 | -5 | *Betula* |
| Loch of Winless | 58.46667 | -3.2 | *Betula* |
| Lochan an Druim | 58.467 | -4.7 | *B.* cf. *nana* |
| Yesnaby | 59.017 | -3.333 | *B. nana* |

**Table S3. Genetic diversity parameters of 55 populations of the three *Betula* species based on microsatellites.**

| Population locality | Latitude | Longitude | N | A | A’ | H_O_ | H’ | F_IS_ |
| --- | --- | --- | --- | --- | --- | --- | --- | --- |
| ***B. nana*** |  |  |  |  |  |  |  |  |
| Ben Loyal | 58.41 | -4.41 | 19 | 77 | 6.42 | 0.57 | 1.55 | 0.08 |
| Ben Wyvis | 57.69 | -4.63 | 15 | 67 | 5.58 | 0.62 | 1.62 | 0.05 |
| Loch Liath | 57.23 | -4.74 | 15 | 66 | 5.50 | 0.58 | 1.55 | 0.17 |
| Inverness | 57.23 | -4.82 | 11 | 75 | 6.25 | 0.63 | 1.58 | 0.04 |
| Braemar | 56.99 | -3.79 | 11 | 61 | 5.08 | 0.54 | 1.53 | 0.10 |
| Desside | 56.92 | -3.20 | 32 | 91 | 7.58 | 0.47 | 1.44 | 0.08 |
| ***B. pubescens*** |  |  |  |  |  |  |  |  |
| Orkney | 58.89 | -3.38 | 23 | 126 | 10.50 | 0.77 | 2.48 | -0.25 |
| Loch Eriboll (E) | 58.49 | -4.66 | 17 | 164 | 13.67 | 0.82 | 2.54 | -0.22 |
| The Crawford | 58.48 | -4.22 | 18 | 152 | 12.67 | 0.82 | 2.58 | -0.26 |
| Tongue | 58.47 | -4.43 | 11 | 150 | 12.50 | 0.82 | 2.56 | -0.21 |
| Benloyal | 58.41 | -4.41 | 74 | 247 | 20.58 | 0.82 | 2.59 | -0.14 |
| Kylesku | 58.25 | -5.02 | 21 | 173 | 14.42 | 0.84 | 2.65 | -0.25 |
| Drumrunie | 57.99 | -5.11 | 8 | 101 | 8.42 | 0.81 | 2.57 | -0.32 |
| btwn Cromarty and Dornoch Firths | 57.80 | -4.24 | 18 | 140 | 11.67 | 0.81 | 2.42 | -0.32 |
| Braemore | 57.76 | -5.03 | 17 | 167 | 13.92 | 0.84 | 2.57 | -0.22 |
| Urqhart Castle | 57.32 | -4.45 | 15 | 168 | 14.00 | 0.85 | 2.65 | -0.34 |
| Lynemore | 57.28 | -3.55 | 21 | 166 | 13.83 | 0.84 | 2.68 | -0.21 |
| Blairnamarrow | 57.22 | -3.30 | 12 | 127 | 10.58 | 0.81 | 2.53 | -0.19 |
| Glen Muick | 57.00 | -3.08 | 19 | 165 | 13.75 | 0.85 | 2.61 | -0.21 |
| N side of Lock Muick | 56.93 | -3.18 | 27 | 156 | 13.00 | 0.80 | 2.52 | -0.19 |
| NW side of Ben Gulabin | 56.83 | -3.49 | 21 | 156 | 13.00 | 0.87 | 2.61 | -0.31 |
| Loch Linnhe | 56.76 | -5.19 | 13 | 136 | 11.33 | 0.82 | 2.48 | -0.25 |
| Rannoch Moor | 56.62 | -4.77 | 23 | 157 | 13.08 | 0.90 | 2.67 | -0.38 |
| Crianlarich | 56.38 | -4.64 | 21 | 171 | 14.25 | 0.84 | 2.58 | -0.25 |
| Bankhead Moss | 56.28 | -2.90 | 20 | 139 | 11.58 | 0.91 | 2.79 | -0.25 |
| Callander | 56.25 | -4.28 | 12 | 149 | 12.42 | 0.84 | 2.55 | -0.23 |
| Loch Lomond | 56.23 | -4.70 | 12 | 139 | 11.58 | 0.85 | 2.57 | -0.31 |
| Roseberry Topping | 54.51 | -1.11 | 12 | 133 | 11.08 | 0.85 | 2.60 | -0.27 |
| BirkPark, Yorks | 54.39 | -2.00 | 19 | 164 | 13.67 | 0.86 | 2.54 | -0.26 |
| ScoutCamp, Lancs | 53.80 | -2.41 | 14 | 113 | 9.42 | 0.90 | 2.73 | -0.36 |
| GlossopWood, Derby | 53.43 | -1.95 | 16 | 125 | 10.42 | 0.82 | 2.51 | -0.24 |
| Pretty Corner, Sheringham | 52.93 | 1.20 | 19 | 176 | 14.67 | 0.89 | 2.63 | -0.26 |
| Cockshood Broad | 52.69 | 1.46 | 20 | 162 | 13.50 | 0.89 | 2.64 | -0.27 |
| Hargham Heath | 52.49 | 0.99 | 15 | 138 | 11.50 | 0.87 | 2.53 | -0.30 |
| RytonWood, Warwick | 52.35 | -1.45 | 14 | 139 | 11.58 | 0.89 | 2.69 | -0.27 |
| BreconBeacons2 | 51.85 | -3.18 | 20 | 146 | 12.17 | 0.80 | 2.51 | -0.21 |
| Danbury Wood | 51.71 | 0.57 | 19 | 137 | 11.42 | 0.84 | 2.41 | -0.33 |
| Widehurst Wood, Marden | 51.15 | 0.49 | 16 | 166 | 13.83 | 0.90 | 2.72 | -0.28 |
| East Hampshire Hanger | 51.11 | -0.93 | 15 | 150 | 12.50 | 0.86 | 2.52 | -0.27 |
| Worth Forest, nr Crawley | 51.09 | -0.16 | 19 | 173 | 14.42 | 0.88 | 2.66 | -0.29 |
| Ebernoe Common | 51.04 | -0.61 | 17 | 158 | 13.17 | 0.84 | 2.42 | -0.20 |
| Byworth nr Petworth | 50.92 | -0.48 | 17 | 158 | 13.17 | 0.85 | 2.54 | -0.24 |
| Beaulieu, New Forest | 50.85 | -1.45 | 21 | 172 | 14.33 | 0.87 | 2.53 | -0.25 |
| DartmoorBog | 50.52 | -3.81 | 13 | 132 | 11.00 | 0.90 | 2.44 | -0.33 |
| DartmoorBurrator | 50.49 | -4.05 | 18 | 165 | 13.75 | 0.88 | 2.66 | -0.28 |
| ***B. pendula*** |  |  |  |  |  |  |  |  |
| Urqhart Castle | 57.32 | -4.45 | 13 | 87 | 7.25 | 0.63 | 1.60 | 0.15 |
| Rinabaich | 57.05 | -3.15 | 9 | 76 | 6.33 | 0.56 | 1.55 | 0.26 |
| ConsettWood | 54.83 | -1.90 | 12 | 75 | 6.25 | 0.63 | 1.59 | 0.10 |
| Pretty Corner, Sheringham | 52.93 | 1.20 | 9 | 68 | 5.67 | 0.63 | 1.59 | 0.15 |
| GulletWood, Worcs | 52.04 | -2.35 | 15 | 86 | 7.17 | 0.65 | 1.62 | 0.07 |
| Brecon Beacons | 51.81 | -3.05 | 16 | 83 | 6.92 | 0.56 | 1.50 | 0.24 |
| Ebernoe Common | 51.04 | -0.61 | 9 | 63 | 5.25 | 0.63 | 1.56 | 0.18 |
| Perry Wood, New Forest | 50.82 | -1.53 | 13 | 76 | 6.33 | 0.58 | 1.51 | 0.19 |
| DartmoorBog | 50.52 | -3.82 | 8 | 76 | 6.33 | 0.47 | 1.65 | 0.10 |
| Dartmoor | 50.52 | -3.80 | 12 | 57 | 4.75 | 0.64 | 1.56 | 0.06 |

N: number of individuals from each population; A: total number of alleles seen across all loci; A’: number of alleles per locus per population, averaged over loci; Ho: proportion of observed heterozygosity averaged over loci. H’: Number of alleles averaged over loci; F_IS:_ (H_E_ - H_O_)/H_E._

Figure S1


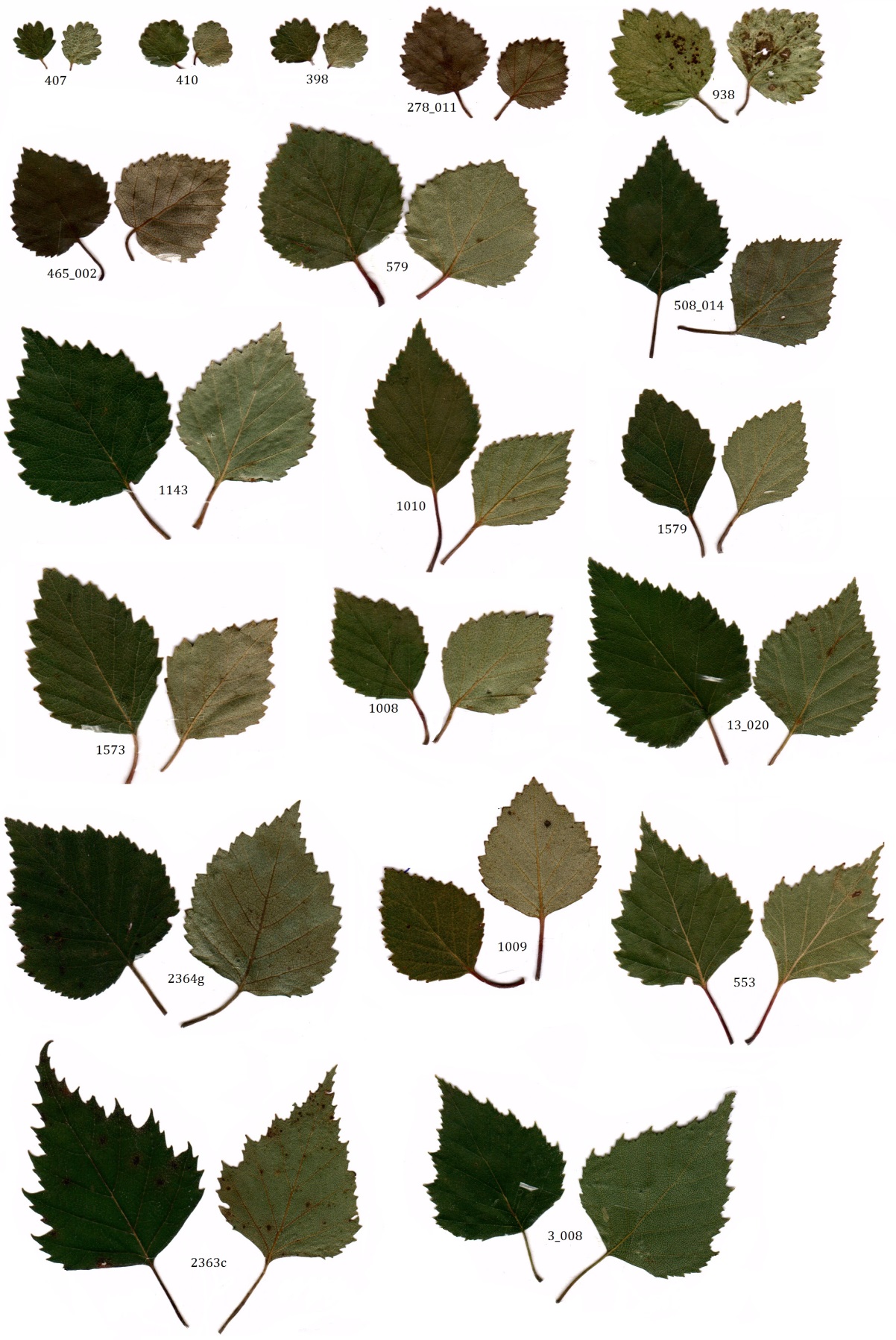


Figure S2


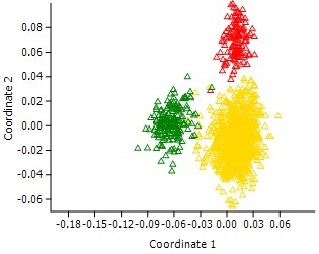


Figure S3


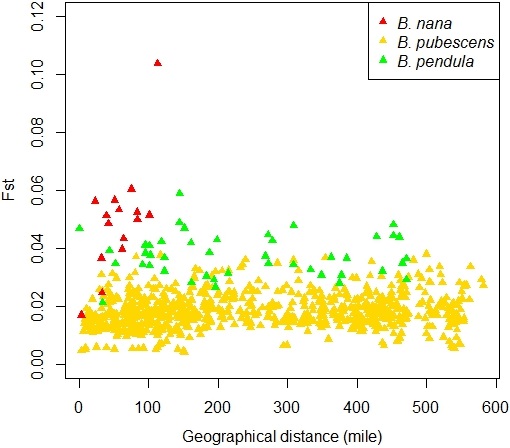


Figure S4


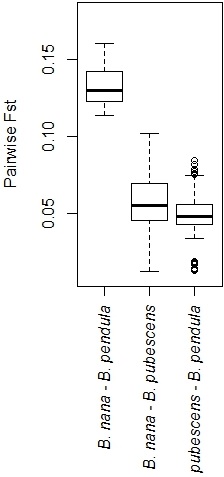


Figure S5


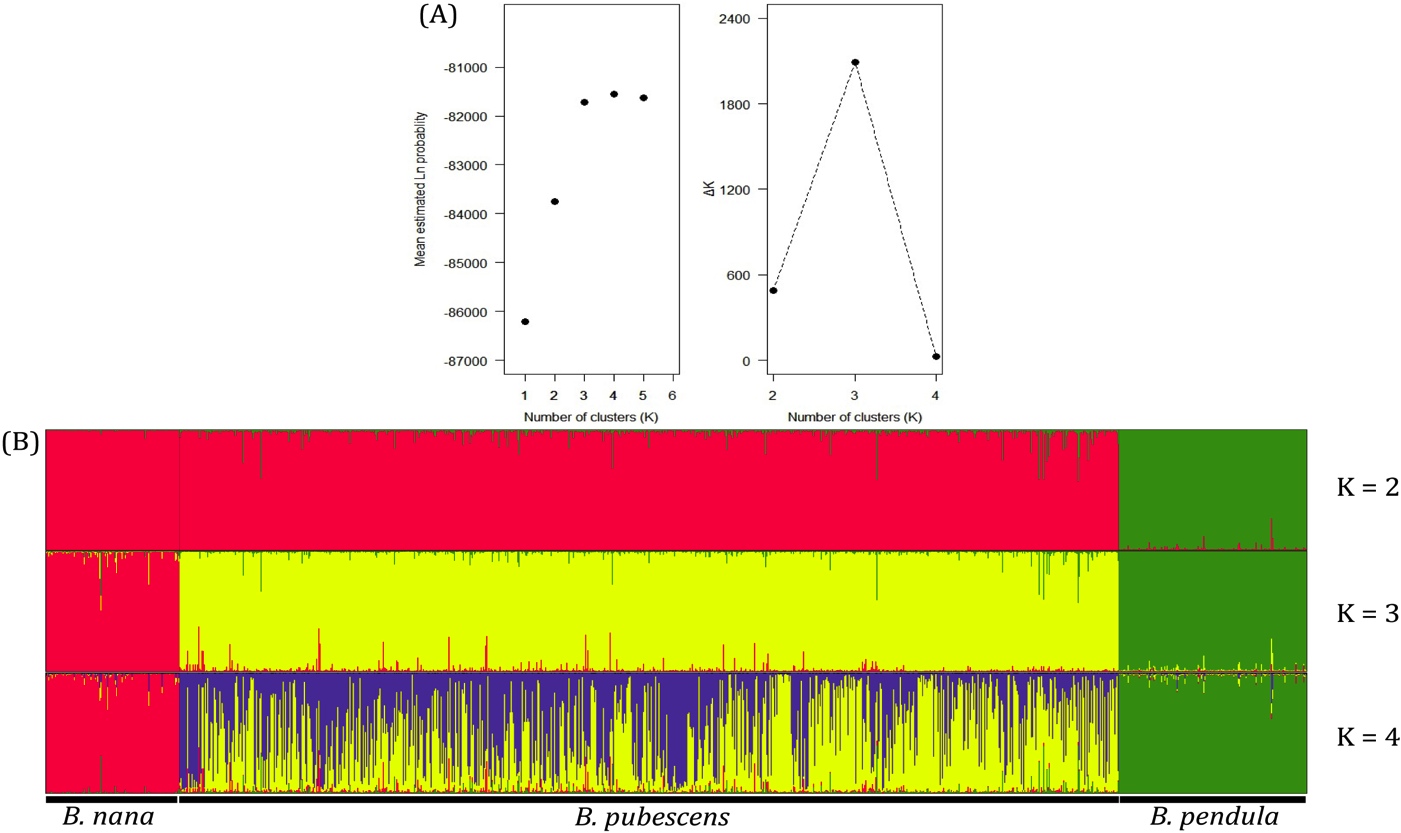


Figure S6


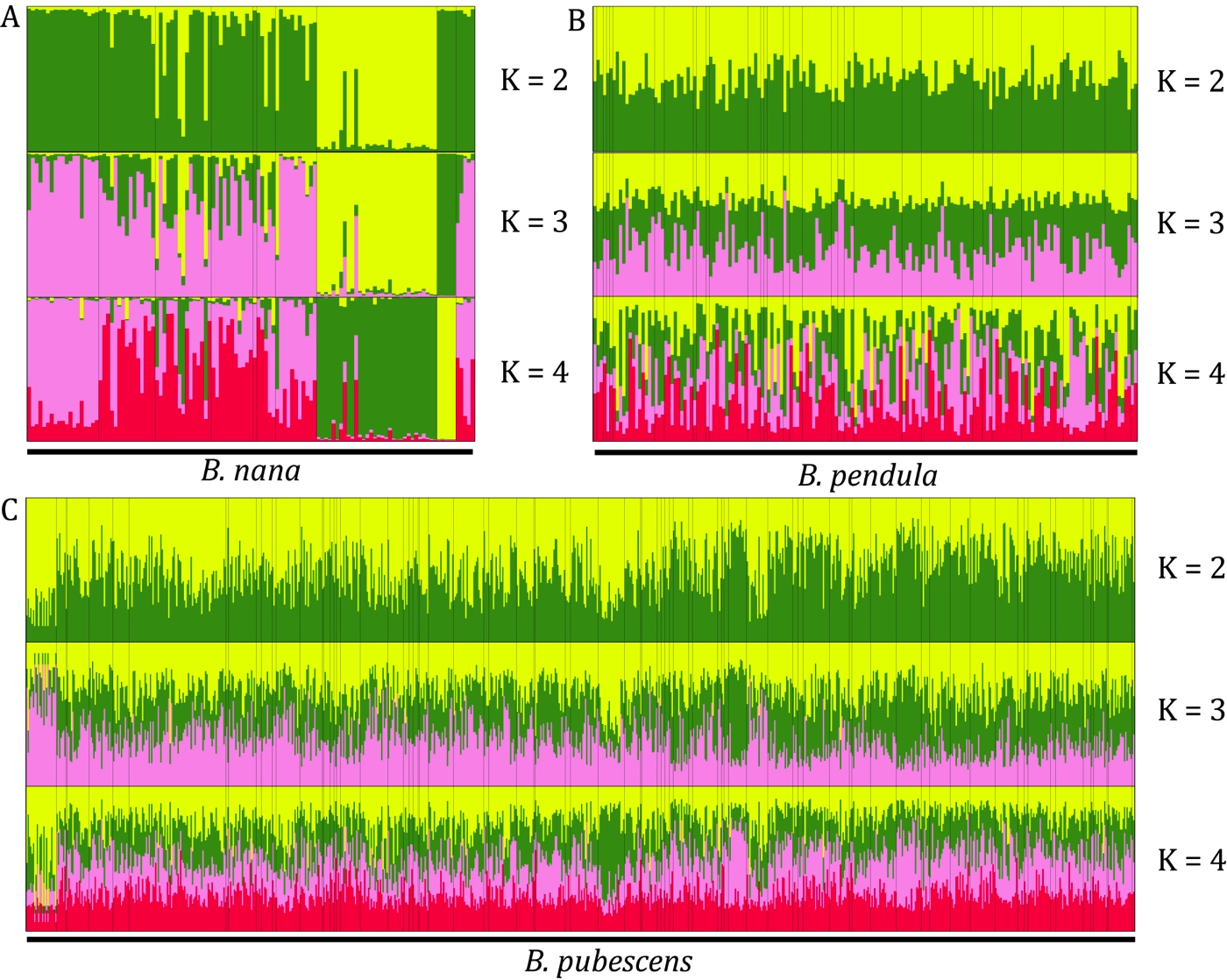

Supplement: Table S1 — Summary of the 16 polymorphic microsatellite loci multiplexed for all samples. Table S2 Summary of published pollen records of Betula species in the UK. Table S3 Genetic diversity of 55 populations with over seven samples of the three Betula species, based on microsatellites. Fig. S1 Pairs of leaves from a representative sample of trees used in the present study, showing upper and lower sides. Fig. S2PCO analysis based on Euclidean distance. Fig. S3 Pairwise genetic differentiation (FST) within species. Fig. S4 Pairwise genetic differentiation (FST) between B. nana,B. pubescens and B. pendula. Fig. S5 (A) The log-likelihood values of each K and ΔK values. (B) The structure output at K = 2, 3 and 4. Fig. S6 The structure output of B. nana,B. pubescens and B. pendula separately, at K = 2, 3 and 4, based on the admixture model. [file mec0023-2771-SD1.docx]
